# Structure determination of a human virus by the combination of cryo-EM and X-ray crystallography

**DOI:** 10.1007/s41048-016-0027-2

**Published:** 2016-09-02

**Authors:** Zheng Liu, Tom S. Y. Guu, Jianhao Cao, Yinyin Li, Lingpeng Cheng, Yizhi Jane Tao, Jingqiang Zhang

**Affiliations:** 1 Department of Biophysics, Health Science Centre, Peking University, Beijing, 100191 China; 2 Department of BioSciences, Rice University, Houston, TX 77005 USA; 3 School of Life sciences, Sun Yat-sen University, Guangzhou, 510275 China; 4 School of Life Science, Tsinghua University, Beijing, 100084 China; 5 State Key Laboratory of Organ Failure Research, Institute of Antibody Engineering, School of Biotechnology, Southern Medical University, Guangzhou, 510515 China; 6 Department of Biochemistry and Molecular Biophysics, Columbia University, New York, 10032 USA

**Keywords:** Structure determination, Cryo-EM, X-ray crystallography, Human virus

## Abstract

Virus 3D atomic structures provide insight into our understanding of viral life cycles and the development of antiviral drugs. X-ray crystallography and cryo-EM have been used to determine the atomic structure of viruses. However, limited availability of biological samples, biosafety issues due to virus infection, and sometimes inherent characteristics of viruses, pose difficulties on combining both methods in determining viral structures. These have made solving the high resolution structure of some medically important viruses very challenging. Here, we describe our recently employed protocols for determining the high-resolution structure of the virus-like particle of hepatitis E virus (HEV), a pathogen of viral hepatitis in human. These protocols include utilizing recombinant baculovirus system to generate sufficient amount of virus particles, single-particle cryo-EM to get an intermediate resolution structure as a phasing model, and X-ray crystallography for final atomic structure determination. Our protocols have solved the hepatitis E virus structure to the resolution of 3.5 Å. The combined methodology is generally applicable to other human infectious viruses.

## Introduction

X-ray crystallography and cryo-EM are two main techniques for visualizing virus 3D structures. One prerequisite for both methods is sufficient amount of samples. For some viruses, samples are purified either directly from their hosts or from tissue culture. Unfortunately, for some viruses including hepatitis E virus, neither sources provide adequate biological sample. In addition, poor infectivity of the virus and biosafety problems further complicate their structural study. In such cases, an efficient in vitro system for generating its substitute, recombinant virus-like particles (VLPs) (Liu et al. 2016), became necessary. The assembled HEV VLPs were expressed without its complete viral genetic materials, therefore bypassing the biosafety issues. Hepatitis E virus (HEV), discovered by immune electron microscopy (Balayan et al. 1983), is the sole member of the genus *Herpesvirus* within the family *Hepeviridae*. It causes acute viral hepatitis in human. Neither stools of the infected patients nor cell culture were able to produce enough sample. Thus, recombinant systems were developed to provide materials for this investigation. The HEV virus-like particles were first obtained by the use of baculovirus insect system expressing the aa112–608 fragment of open reading frame 2 (ORF2) (Li et al. 1997), and then 3D structures of both *T* = 1 and *T* = 3 particles were solved at low resolution using cryo-EM (Xing et al. 2010). When using *E. Coli* to express the HEV capsid protein, only P2 particles were assembled by expressing the protein of aa394–606 (Li et al. 2009). The P2 particles and their complexes with monoclonal antibodies were subsequently determined by X-ray Crystallography (Tang et al. 2011). The high-resolution atomic structure of HEV VLP were eventually determined by several laboratories independently (Guu et al. 2009; Yamashita et al. 2009; Liu et al. 2011).

Here, we present our protocol for determining high resolution of the HEV VLP by combining EM and X-ray crytallography techniques. The protocol, utilizing recombinant baculovirus system to obtain sufficient amount of virus particles, and single-particle cryo-EM to get an intermediate resolution structure as a phasing model, and then X-ray crystallography for final structure determination, solved the hepatitis E virus structure to the resolution of 3.5 Å. The high-resolution structure reveals intricate details in HEV-VLP, including three linear domains—S, P1, and P2—arranged in a manner different from caliciviruses, which also possess the three domains. This combined approach can also be applied to structural investigation of other infectious viruses with low abundance in nature, high infectivity, and other unique structural features, which may make it challenging to study using either cryo-EM or X-crystallography alone.

## Overview of experimental design

**Fig. 1:**
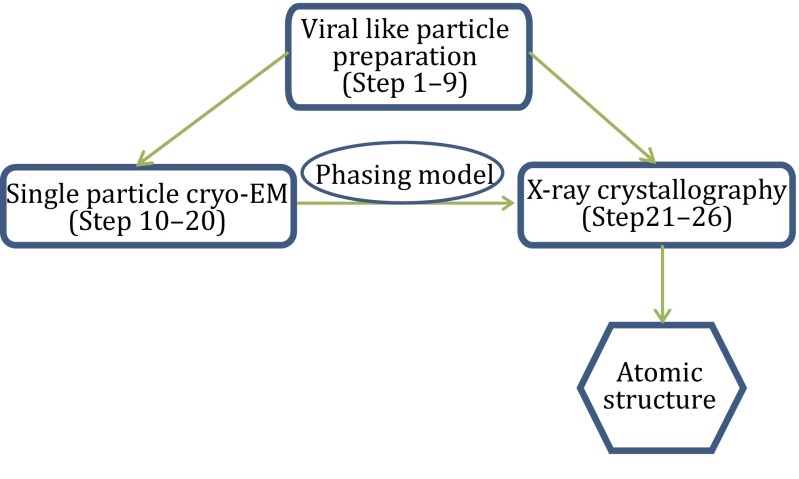
The flowchart for the determination of the HEV virus-like particle structure using the baculovirus insect system, single-particle cryo-EM, and X-ray crystallography

**Fig. 2:**
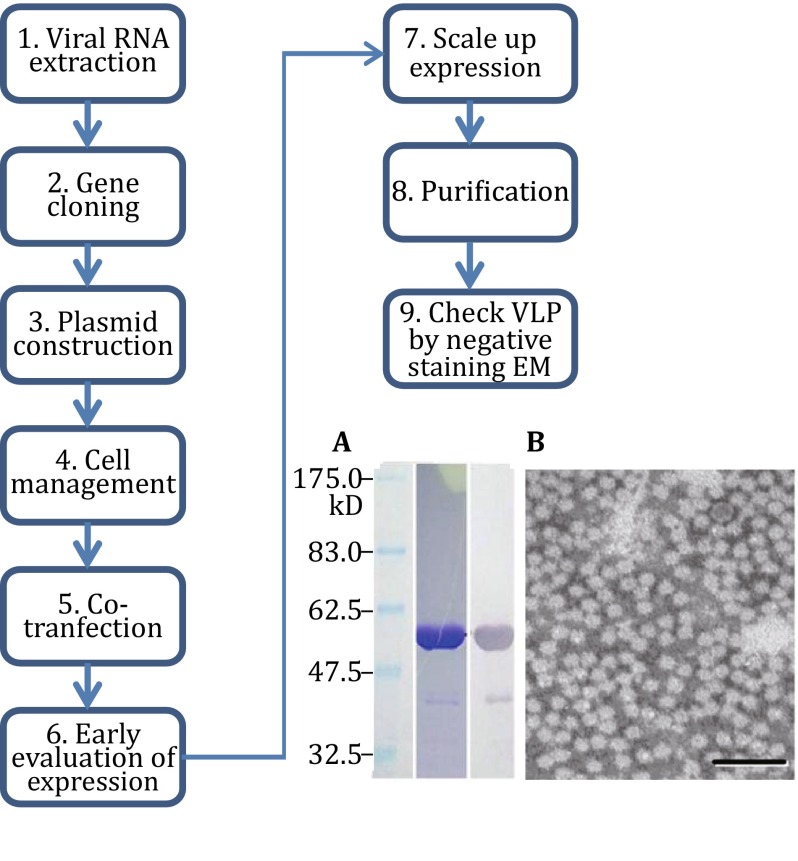
The HEV viral capsid protein expression and virus-like particle assembly. An outline of the procedures used for the expression and purification of HEV capsid protein is shown. The results of protein SDS-PAGE electrophoresis, Western blotting, and particle observation by negative stain microscopy are shown on the *right*. The scale bar in **B** is 100 nm

**Fig. 3:**
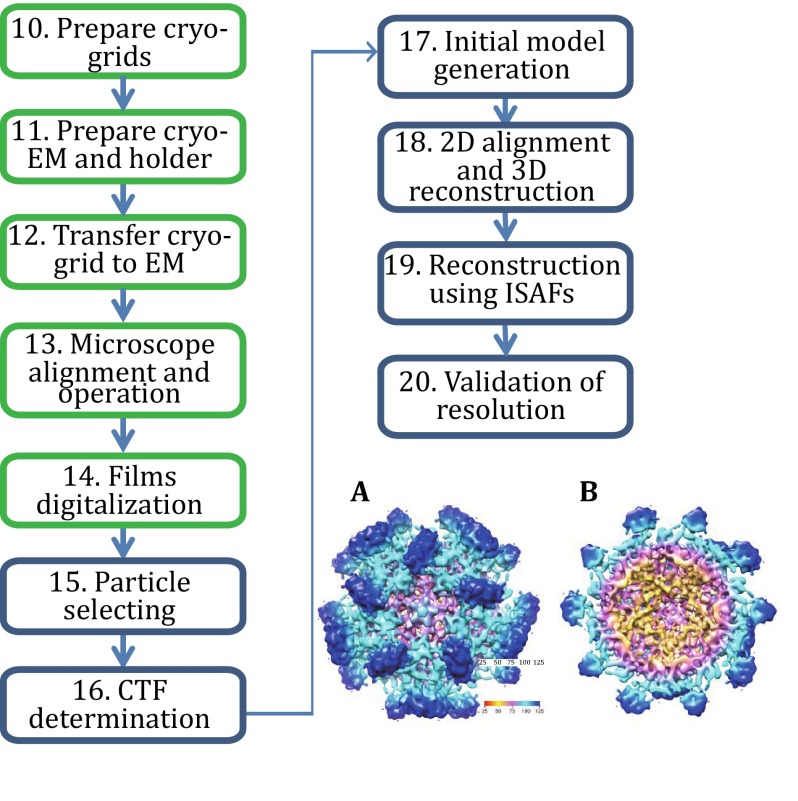
The cryo-EM and image processing of 3D structure of hepatitis E virus. An outline of the procedure used for the single-particle analysis of HEV virus-like particle is shown. Steps involving the cryo-EM are highlighted in *green*, whereas steps involving the image processing are shown in *blue*. The reconstruction at 9.8-Å resolution is shown on the *right*. **A** The density map is viewed along the 5-fold axis. **B** The internal view of the virus-like particle

**Fig. 4:**
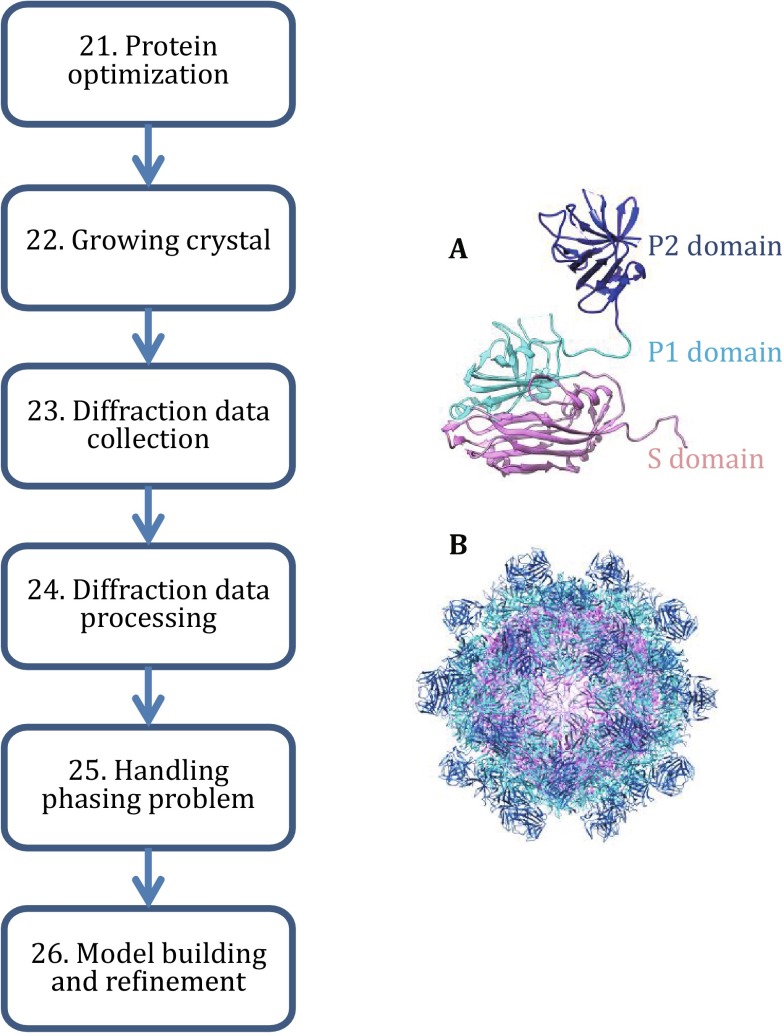
The X-ray crystallography of virus-like particle of hepatitis E virus. The procedures to obtain 3.5-Å resolution structure are shown. **A** The monomer of the HEV capsid protein is shown on the *right*. **B** The whole model of the HEV virus-like particle viewed from along the 5-fold axis

Our protocol can be grouped into three sections (Fig. 1). The first section (Step 1–9) describes the procedures to generate sufficient and suitable virus-like particles for single-particle cryo-EM study (Fig. 2). The second section (Step 10–20) details the steps of performing single-particle cryo-EM to get a 3D density map (Fig. 3). The third section (Step 21–26) encompasses the details of using X-ray method to yield the final high-resolution structure (Fig. 4).

In the first part of the protocol, we will provide an overview on how to purify the virus gene, to construct recombinant plasmid, to scale-up expression, and to carry out purification. Since making recombinant plasmids and preparing cell culture involve multiple detailed steps, it is strongly recommended to follow manufacturers' manuals.  

In the second set of steps, we describe the details of how to freeze cryo-EM sample, to operate transmission electron microscope, and to perform image processing. Once the micrographs are collected, the particles of the virus are picked out. The images of particles are then assigned with a group of positional parameters including translation and orientation information by refining an initial model. The main image processing procedures are performed using programs from EMAN (Ludtke et al. 1999) and IMIRS package (Liang et al. 2002).

In the final section, the crystallization and data processing are detailed. For obtaining the phase information, cryo-EM model from the second step is used as an initial phasing model.

## Materials and equipment

### Materials and reagents for producing virus-like particle

DNA purification kits, PCR kit, *E. coli* DH5α, and TOP10 strain. Cell lines and cell culture media: sf9, sf21, high five cells; TC-100 media, EX-CELL 405 media (Sigma-Aldrich, USA), Grace Media, tissue culture flasks, BD BaculoGold linearized baculovirus DNA (BD Bioscience, USA), Cellfectin reagent (Invitrogen, USA), transfer vector (Pharmingen, San Diego, USA).

### Equipment for Cryo-EM

SO163 films (Kodak, USA); D19 (for low dose film development) (Kodak, USA).

JEM-2010 electron microscope (JEOL, Japan) equipped with a Gatan 626 Cryo-holder (Gatan, USA), R2/1, 200-mesh holey grids (Quantifoil, Germany), ion coater (Eiko Engineering Co., LTD), model 655 pumping station (Gatan, USA), home-made plunge freezer, and LS-8000ED scanner (Nikon, Japan).

### Software packages for cryo-EM reconstruction

EMAN (Ludtke et al. 1999), IMIRS (Liang et al. 2002).

### Software packages for X-ray crystallography

General locked rotation function (GLRF) (Tong and Rossmann 1997), MAVE (Read and Kleywegt 2001), MAPROT (Stein et al. 1994), MAPMAN (Kleywegt and Jones 1996), SFALL (Ten Eyck 1977), RSTATS (Collaborative 1994), AVE and RAVE (Jones 1992), O (Jones et al. 1991), CNS (Brunger et al. 1998).

## Summarized procedure

1. Viral RNA extraction is performed on the stools from the patients of hepatitis E;
2. The ORF2 of hepatitis E virus is cloned into TA vector;
3. The target gene is inserted to the plasmid for transfection;
4. The sf9 and high five cells are cultured for transfection and expression;
5. The transfecting plasmid with interest gene is used to transfect sf9 cells;
6. Early evaluation of the HEV capsid protein expression is performed in P1 cells, which is then followed by baculovirus amplification;
7. Scale up expression using high five cells and high-titer baculoviruses;
8. The virus-like particle is purified using ultracentrifugation;
9. The VLP sample is checked by negative staining EM;
10. Prepare cryo-grids;
11. Cool down EM and holder;
12. Transfer cryo-grid to the column of the electron microscope;
13. Microscope alignment is careful operated;
14. Films digitalization using Niko scanner;
15. Viral particles are selected using EMAN boxer;
16. CTF determination is performed by CTFIT;
17. Initial model generation using minimum phase residue method;
18. 2D alignment and 3D reconstruction for individual particle images;
19. Reconstruction of all the aligned particle images using ISAFs;
20. Validation of resolution;
21. Protein preparation for crystallization;
22. Growing crystal;
23. Diffraction data collection;
24. Diffraction data processing;
25. Handle phasing problem;
26. Model building and refinement.

## Procedure

### Capsid protein expression and VLP purification

#### Viral RNA extraction

The gene of target proteins of infectious viruses is usually obtained from various sources: (1) clinical samples including throat swab, stool, body fluids, etc.; (2) synthesized gene by commercial company; (3) gifted plasmid containing the gene of interest. To obtain viral gene, in this study, stool sample was first collected from patient with confirmed hepatitis E (non-A, non-B acute hepatitis) infection. The stool sample was preserved at −80 °C for less than two weeks before being subjected to RNA extraction.

The stool sample was suspended with 4×volume of phosphate-buffered saline and then the mixed specimen was homogenized by vortexing for 1 min. The resulted mixture was subjected to centrifugation steps with a speed of about 1500 RCF first and the supernatant was applied to another centrifugation with 12,000 r/min at 4 °C for 20 min to further remove the sediment. The total RNA extraction from the pretreated stool sample was performed using commercial RNA extraction kit. The extracted product was stored at −80 °C for the downstream usage including PCR.

#### Gene cloning

This step aims to amplify the target gene and then to transfer it into a proper vector or plasmid. Amplification of the entire HEV ORF2 by PCR was performed with the following primers:

- HEV-D2 (59-TGGGTTCGCGACCATGCGCCCTCG-39),
- HEV-U2 (59-CAACAGAAAGAAGGGGGGCACAAG-39).

The PCR product was first examined by DNA gel, and then was subjected to gene sequencing. After obtaining the PCR product with accurate sequence of the ORF2, we used TA cloning to transfer the PCR products using TA Cloning Kit with PCR 2.1 vector (Invitrogen, USA).

#### Plasmid construction

Target genes were cloned into a transfer vector which was used to manipulate the viral genome. Here, we used PVL1391 based on homologous recombination.

(A) The T vector with HEV-ORF2 gene was digested with *NruI* and *XbaI*, and the resultant 2-kb fragment was ligated with a transfer vector pVL1393 digested with *SmaI* and *XbaI*.
(B) Set up a DNA ligation to fuse the digested pVL1393 vector and the cDNA fragment. Typically 100 ng of the linear plasmid fragment was ligated with three-fold molar excess of the insert in 10 μL reaction volume.
(C) Transformed the ligation reaction into competent cells, for example *E. coli* DHα5 and plated onto LB agar plates containing 100 μg/mL ampicillin.
(D) Checked the transfer vector if it contained the gene of choice. Picked up 4–5 colonies and grew 1 mL precultures and then used the HEV-D2 and HEV-U2 primers for PCR analysis to check target gene.
(E) Precipitated 10 μg DNA with 300 mmol/L Na/Acetate pH 5.2 (final concentration) and three volumes of ethanol 100%. Resuspended DNA in 20 μL sterile ultrapure H_2_O. Took an aliquot to measure the DNA concentration and stored at −20 °C.

[**CAUTION!**] Site-specific transposition and cotransfection. For generating recombinant baculoviruses, there are two different available methods. Both methods make use of the baculovirus genome engineered into a bacterial plasmid, which was subsequently ampllifed in *E. coli*. The first method is based on site-specific transposition (Tn7 transposition) of an expression cassette into the baculovirus genome in *E. coli*; the second employs a transfer vector and viral DNA that are cotransfected into insect cells and utilizes host enzyme-mediated homologous recombination.

#### Cell management

**Fig. 5:**
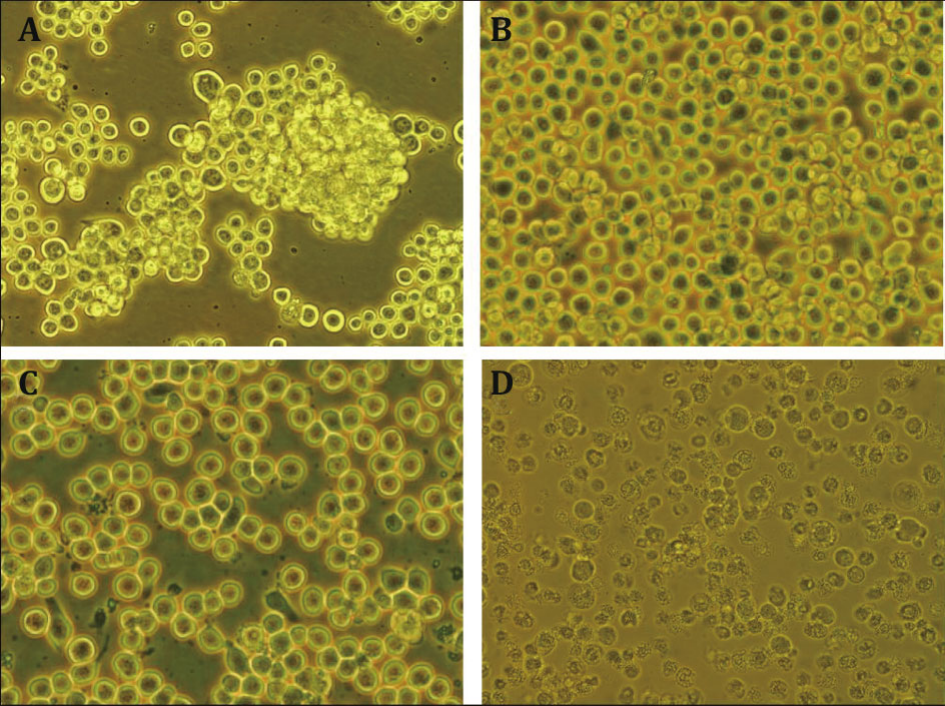
Cell management of sf9 insect cell line. **A** The aggregated cells showed suboptimal status. **B** The cells achieved 90% confluence indicating a time point for passage. **C** Cells for infection of viral stock. **D** Cell debris after 4-day postinfection
vi. Took an aliquot of the cell suspension, counted cells, and determined their viability.
vii. Added an appropriate volume of medium to the flask and aliquotted the cells into three new flasks with a starting density of 0.5 × 10^6^ cells/mL and incubated cells at 27 °C.

In general, insect cells are handled in a laminar flow hood under aseptic conditions. All cell culture experiments are carried out at 27 °C. The basic work on cells is classified into three steps: cell recovery, cell passage, and cell storage.

(A) Cell recovery.

i. Removed vial of cells from liquid nitrogen and placed in water bath at 37 °C. Thawed them rapidly in the water bath.
ii. Decontaminated the outside of the vial by spraying with 70% ethanol.
iii. Transferred the 1 mL thawed cell suspension directly into the 4 mL of media, and then incubated flask at 27 °C and allowed cells to attach for 30–45 min.
iv. After cells were attached, removed the medium, and fed cells with 5 mL of fresh medium. After 24 h, exchanged with fresh medium.

(B) Cell passage.

v. When cells grew until 90% confluence (Fig. 5), removed the growing culture and detached cells by tapping the flask.

(C) Cell storage.

viii. Prepared cryo-vials, cool them on ice. Centrifuged cells at 100–150 *g* for 10 min at RT and removed supernatant.
ix. Resuspended cells at the proper density. Then, transferred 1 mL to each sterile cryo-vial.
x. Placed the cells at −20 °C for 1 h, then stored at −80 °C for 24–48 h. Transferred them to Dewars filled with liquid nitrogen for long-term storage.

#### Cotransection

To generate recombinant baculoviruses, the transfer vector is cotransfected with linearized viral DNA in insect cells.

(A) Seeded a 6-well plate using 1.5 × 10^6^ sf9 cells per well in 1.6 mL insect cell culture medium (TC100 + 10% FBS) and let the cells adhere for 20 min at 27 °C (Fig. 5C). Meanwhile, mixed 4 μg of DNA transfer vector with 1 μg of linearized DNA in 100 μL of sterile 150 mmol/L NaCl.
(B) Added 200 μL DNA/cell-transfection solution drop to the cells, and incubated at 27 °C. Four hours after the cotransfection, added 2 mL of insect cell medium to the cell layer and returned to the incubator for at least 5 days.
(C) From the second day, observed cells daily by an inverted microscope and searched for infected cells. After 5 days, carefully collected the supernatant by centrifugation at 200 *g* for 10 min (P0) and stored  at 4 °C.
  The cotransfection product is referred to as the initial virus stock (P0). It can be used for further evaluation of the target protein expression and for large-scale expression.

#### Early evaluation of protein expression

P0 can be used for an initial screening to provide the first indication of the expression level.

(A) Seeded a 6-well plate using 1.5 × 10^6^ sf9 cells per well and let the cells adhere for 20 min at 27 °C. Discarded the medium; added 300 μL of fresh medium and 150 μL of P0 to attached cells followed by 1 h incubation at 27 °C.
(B) Added 3 mL of insect cell medium and returned to the incubator for 48 h. Collected the infected cells by centrifuging at 200 *g* for 10 min at 4 °C.
(C) Resuspended cells in 1 mL of lysis buffer containing 1% Tween20 and 400 U/mL of DNase Type I with EDTA-free protease inhibitor and sonicated them for 30 s. Took 15 μL aliquots and add 5 μL of 4× SDS loading buffer.
(D) Analyzed the different samples using SDS-PAGE with coomassie staining and/or with western blotting in case of low expression levels.

#### Scale up expression of protein

There are two common strategies to scale up the expression of protein: one uses cells grown in suspension in the Fermentor/Bioreactor and the other uses large tissue flasks with adherent culture. We used the second method to scale up our protein expression.

To ensure the viral stock is of high infectious efficiency, one (P1) or two (P2) or even more round of amplification can produce virus stocks of high titers. The yield of the recombinant protein is affected by a number of factors. Thus, once a concentrated baculovirus stock has been made, optimization experiments are required before large scale expression. In general, the optimization is performed for two variables: the amount of infectious virus and the best time for harvesting. In our HEV expression experiment, we used the MO1 at 10 and harvested our expressing cell after 3 days of postinfection.

#### Purification of virus-like particle

Instead of using FPLC, we used gradient ultracentrifugation for HEV-VLP purification.

(A) Infected cells and media were pelleted at 10,000 *g* for 90 min and the pellet was discarded.
(B) The supernatant was pelleted again using Beckman rotor SW28 at 25,000 r/min at 4 °C for 2 h. The resulting pellet was resuspended in 4.5 mL PBS at 4 °C overnight.
(C) The resuspended pellet was mixed with 1.95 g CsCl and centrifuged by using rotor SW55Ti at 35,000 *g* at 4 °C for 24 h.
(D) The tube containing CsCl mixed with virus-like particles was punctured with a needle to collect fractions of different densities. Each fraction was diluted with PBS and dialyzed against PBS to remove CsCl.
(E) After removing the CsCl, the sample was subjected to concentration check. For long-term storage, the sample was aliquoted and stored at −20 °C.

#### Check VLP by negative staining EM

To evaluate the overall quality of our HEV-VLP samples, negative staining was implemented.

(A) About 3 μL of purified sample was applied to a newly glow-discharged grid coated with carbon film.
(B) After about 2 min adsorption, the sample on the grid was blotted using a filter paper.
(C) A droplet of 2% phosphotungstic acid was applied to the grid, and after 1 min, the stain was wicked away again using a filter paper.
(D) The grid was dried in air and examined under TEM operated at 120 kV.

### Single-particle cryo-EM

#### Preparation of cryo-grids

Once the sample was deemed adequate for cryo-EM, the next step was to prepare cryo-EM grids. To make an optimized grid with thin ice and good particle distribution, it was a trial-error procedure to find out optimal blotting time and blotting force. In general, preparation of cryo-EM grids was carried out in four steps.

(A) Cleaned grids. In order to remove any residual polymer on the grids, they were subjected to washing with chloroform.
(B) Treated grids. For samples that prefer to stay at the hole edge of the grid, one can evaporate carbon on the holey grid to coat a thin layer of carbon film over the surface of the grid. This step is optional.
(C) Glow discharge. The glow discharge converts the naturally hydrophobic carbon-coating layer of the grids into hydrophilic. Placed cleaned grids into the chamber of the glow-discharge equipment and closed the chamber. Pumped the bell jar and glowed discharge for 20 s at 25 mA and 7 × 10^−2^ mbar.
(D) Sample plunge-freezing. Cooled down freezing apparatus by filling up liquid nitrogen. Then ethane was poured slightly into the inner container of the freezer. 4 μL of purified HEV-VLP sample with a concentration of 2.5 mg/mL was applied to a quantifoil R2/1 100 mesh grid. After 1 min, the excessive sample was removed with a filter paper. The prepared grids were transferred into liquid nitrogen Dewars for storage.

#### Prepare EM and cryo-holder

Before loading the sample to the column of microscope, some preparation was needed for the instrument and for loading the sample.

(A) Checked the EM status. Read the logbook and checked if the previous session was normal. Ensure the cryo-cycle was finished before starting this new session.
(B) Checked the column vacuum (<3 × 10^−5^ Pa) and the gun vacuum (0.1 × 10^−6^ Pa). Made sure the high tension (HT) is ready.
(C) Filled up the anti-contamination device of the microscope with LN2. Made sure the stage is centered.
(D) Prepared cryo-holder. A cryo-holder is usually stored on a dry pumping station. To start a new cryo-EM session, the cryo-holder was recommended to regenerate the zeolite desiccant, and to achieve a high vacuum in the cryo-holder Dewars.

#### Transfer cryo samples into electron microscope

Loading a cryo-EM grid to the column of microscope is a critical step for cryo-EM. In particular, for 200 kV microscope, this step brings air in to the column and can disrupt the vacuum status. Thus, this step requires much attention when operating the microscope.

(A) Inserted the cryo-holder into the cryo-transfer workstation. Cooled down the workstation by filling with liquid nitrogen.
(B) Plugged the cold stage controller cable to the cryo-holder. Kept monitoring the temperature and added liquid nitrogen if necessary until the temperature stabilized at around −194 °C.
(C) Quickly transferred the cryo-grid box to the workstation. Loaded the grid to the sample holder slot and made sure the clip ring on top of the grid was firmly seated in place. Closed the cryo-shutter, and moved the entire workstation with holder and grid to the microscope console.
(D) Turned the goniometer airlock switch from ‘AIR’ to ‘PUMP.’ Quickly transferred the cryo-EM holder to the stage by aligning the holder airlock pin with the goniometer slot, and slightly pushed it straight in until stopped. When the airlock opened and the green light was on, turned the holder slowly clockwise about 30° until stopped, made sure to hold it firmly so that it was sucked in slowly by vacuum. Continued to turn the holder ~60° clockwise until stopped, and then let it in slowly.
(E) Added liquid nitrogen to the specimen holder Dewar. Waited for the microscope column vacuum to recover (about 1~3 × 10^−5^ Pa). Opened the specimen holder shutter and then opened the gun valve.

#### Alignment and operation of electron microscope

Before starting data collection, the microscope should be carefully aligned to ensure it runs at an optimal state. The following is the detailed protocols for the alignment of our JEM-2010 for the HEV project data collection.

At the beginning, set up the high voltage to 200 kV in the HT ramp-up program, set step to 0.1 kV, time to 30 min, and then started the program.

(A) Aligned the TEM by following these steps in order:

i. Gun alignment. At MAG 50 k, focused the beam (C2), activated the node Wobbler, pressed the GUN Deflection button, adjusted the GUN DEF X and Y to minimize swipe of the illumination disk, and the GUN SHIFT to center the disk.
ii. Condenser alignment. Centered the beam with the GUN SHIFT X and Y at SPOT SIZE 1, and BEAM SHIFT X and Y at SPOT SIZE 5, repeated several times until the beam position was stable when the SPOT SIZE was varied.
iii. Inserted and selected a proper condenser aperture size (e.g., the third one, 40 mm). Centered the aperture by spreading the beam with C2 to half the screen size, centered the illumination disk by adjusting the mechanical X and Y shift of the C2 aperture; focused the beam (C2), and centered the beam with BEAM SHIFT X and Y. Repeated several times.
iv. Voltage centering. This step is to minimize the image movement when the image focus is varied. At a higher MAG of 120 k, centered and focused a recognizable sample feature. Activated the HT WOBBLER and the BRIGHT TILT; adjusted the DEF X and Y so that the feature at the center of the small fluorescent screen remained stationary.
v. Adjusted beam tilt (also called the pivot). At MAG 150 k, focused and centered the beam, activated the CONDEF ADJ TILT button, turned the white toggle switch TILT to X position. This wobbled the beam by modulating the deflection lens current. Used the SHIFT X and the DEF X knobs to form a single spot. Repeated for the TILT Y position.
vi. Corrected the astigmatism of the object lens at a magnification of 600 k.

(B) Imaging at low electron dose.

i. Made sure the temperature was stable at −170 °C.
ii. Opened the filament and checked if the grid was in the “Search Mode.”
iii. Checked the grid quality under “Search Mode” to determine whether it showed evenly particle distribution and thin ice. If so, the grid could be used for data collection.
iv. Shifted to “Focus Mode,” adjusted the z-height of the grid, then focused on an area near the exposure site.
v. Shifted to “Photo/Exposure Mode,” loaded a film and pressed the button “Photo” to expose.

[**Note**] The temperature of the sample had to be under −168 °C throughout all of the procedures.

#### Films development and digitalization

A new type recorder, termed direct detector, is much superior to photographic film and CCD (Liu and Zhang 2014). In the following, we will describe the procedures for developing films which were used during HEV structural study.

(A) Exposed films were first put into a rack for development in batch. Made sure that the room temperature and solution were at 20 °C.
(B) Emerged the rack in Developer for exactly 12 min at 20 °C. Drained excessive solution in <10 s.
(C) Washed the rack in water for 1 min and drained excessive water as much as possible. Emerged the rack in Fixer for 4 min and then drained excessive solution. Washed fixed films with running water for 30–60 min, then air dried them.

In our experiment, we used Nikon LS-8000ED scanner. The scanning condition was set at 4000 dpi with a pixel size of 1.27 Å.

[**CAUTION!**] Recorders of electron microscope. Photographic film and CCD are two traditional recorders for electron microscope. CCD is superior to films considering the convenience of usage. It can output direct digital images without extra workload for development and digitalization that are necessary for films. It also allows for continuous data collection without the need to exchange it and for imaging automation on microscope. Recently, thanks to a new type recorder called electron direct detector device (DDD), cryo-EM field is experiencing revolution. DDD possesses both film and CCD advantages including high DQE and direct outputting digital images. In addition, it allows collecting movies (multiple frames) for each exposure. With image processing, one can correct drift induced by electron interacting with sample, which is a long-standing difficult problem for high-resolution cryo-EM.

[**CRITICAL STEP**] The films with obvious astigmatism, contamination, shift of samples, ice crystals, and few particles were excluded.

#### Boxing particles

**Fig. 6:**
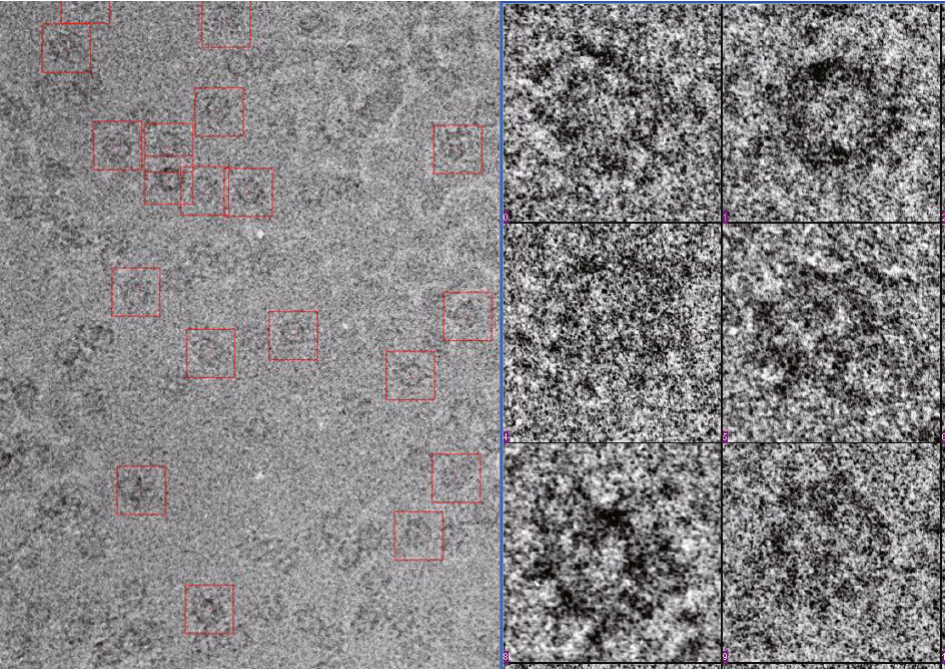
Cryo-EM micrograph of hepatitis E virus particles. *Left*: an area of a micrograph with particles highlighted in *red boxes*. *Right*: six selected particles
(B) All particles are boxed manually. The center of the boxes should be near the center of particles.
(C) All the coordinates of these particles were stored in box files.

To make sure only good quality micrographs were used for further image processing, the first step was to select the particles from micrographs.

(A) The command “boxer” in EMAN software package was used to call the particle-picking module. The box size was set to 256 pixels for HEV-LP selection (Fig. 6).

#### CTF determination

**Fig. 7:**
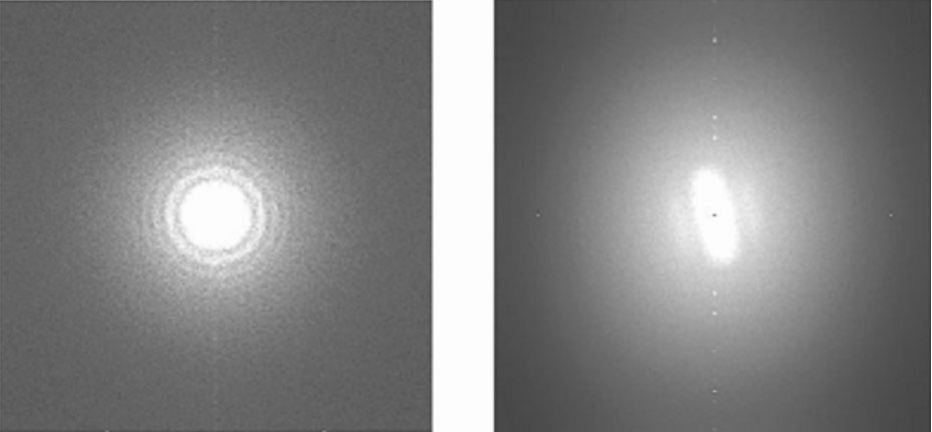
Power spectrums of two representative micrographs. *Left*: power spectrum of a good micrograph displays round thon rings. *Right*: power spectrum of a suboptimal micrograph with drift and astigmatism reveals oval-shaped thon rings

Among many of the parameters (defocus, B-factor, noises, etc.) of the contrast transfer function (CTF), the defocus value is the most critical parameter (Jiang et al. 2012). In addition to determining the CTF parameters, the Thon rings in power spectrum of micrographs can be used for evaluating the quality of micrographs. The circular Thon rings indicate good quality images regardless of other factors; non-circular Thon rings show astigmatisms (Fig. 7).

This step was performed with ‘ctfit’ in EMAN1.9 software package. The parameters were set as following: accelerate voltage 200 kV, Cs of EM 2.0 mm, step size of images 1.27 A/pixel, contrast amplitude of images 0.1.

#### Reconstruct initial model

**Fig. 8:**
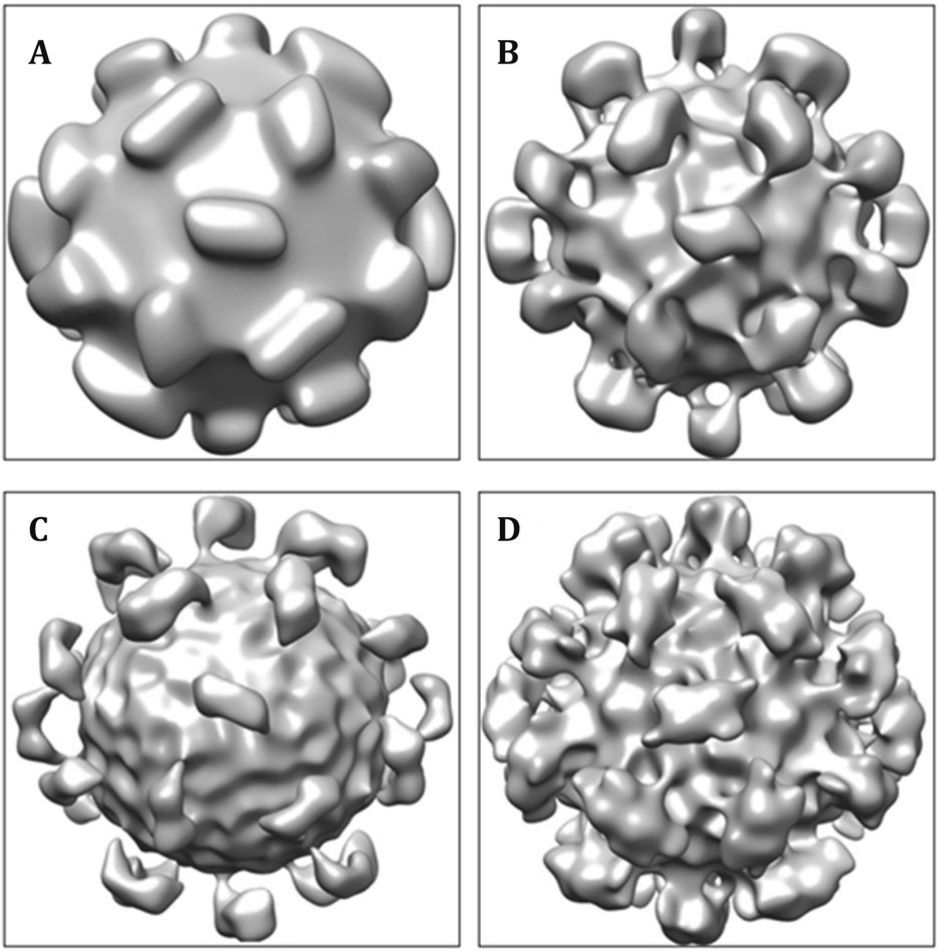
Four initial models reconstructed from different groups of particles. The map showing a consistent icosahedral spike pattern was the most promising initial model

Three-dimensional (3D) reconstruction of a pool of particle images requires their positional parameters. These parameters were initially assigned by aligning the particle images against an initial model and then iteratively refined by aligning with the reconstructed map from the previous iteration.

(A) Used *ortall* to assign orientation and center of all particles. The parameters were: radius of mask 124, pixel size 1.27 Å/pixel; the maximum and minimum Fourier radius for common line search: minR = 3, maxR = 9.
(B) Selected 7–10 particles with smaller phase residues. Some rules should be followed in picking up particles to build a template. All particles should have similar defocus values and be picked up from the same photograph. The orientation should not be near the 2/3/5-fold symmetrical axes.
(C) From *template.log*, located three particles whose cross-correlation residues were as small as possible, and then used them to compute an initial model. The HEV models used in the study are shown in Fig. 8.

#### 2D alignment and 3D reconstruction

Once the initial model has been generated, all the particles’ orientation could be assigned and refined based on this model and later reconstructed maps. Here is the procedure.

(A) Used *eliminateort* to refine orientation and center of all particles with cross-common line phase residue method. The running parameters of *eliminateort* were as follows: threshold of cross-common line phase residue was 55, image size of particles was 256, minimum and maximum value of Fourier radius were 3 and 9. The result of *eliminateort* was stored in file *definedort.dat*.
(B) Reconstructed density map (*reconstruct definedort.dat* *).* Need for CTF correction: yes; diameter for reconstruction (odd): 511. maximum Fourier radius for reconstruction: 45. The *reconstruct* produces three map files: *icos3* *f.map*, *icos2* *f.map*, and *icos5* *f.map*.
(C) Produced projections of 3D density map. Ran command line with projection size 256 and projection step *projecticos_auto icos5f.map ptemps.dat*.
(D) Used the 20 projections above as models and made FFT with the following command: *masktrans ptemps.dat mask 121 prj*. File *ptemps.dat* was a model containing 20 particles projections including orientation and center. 121 was the radius.
(E) Refined orientations and centers of all particles: *refineall* *ptemps.dat*. Some parameters were needed for input.
  Scanning step size: 1.27; minimum and maximum Fourier radius: 3 and 9; the step size for refining orientations of particles: default value; the step size for refining centers of particles: default value; threshold for phase residue: 55.
(F) Made FFT of new orientation and center files: *masktrans newOrt.dat 129 hev*.
(G) Repeated Step C–F until the orientations and centers were stable.

#### Reconstruction using recISAFs

The icosahedral symmetry-adapted functions (ISAFs) reconstruction method was developed by Hongrong Liu et al., which takes advantage of the icosahedral symmetry of virus particles (Liu et al. 2008). It can reconstruct the same set of images to higher resolution when compared to conventional approach. The following was the setting for *recISAFs*: orientation file name was *x.dat*; number of particles was 891; diameter of particle (odd only) was 261. Maximum Fourier radius in reconstruction depended on the procedure; need for CTF correction: yes; memory of the computer (G): 2G. The program produced three map files: *sph3.map*, *sph2.map*, and *sph5.map*.

#### Validation of resolution

In general, the resolution of the 3D reconstruction is determined using the Fourier shell correlation (FSC) between two reconstructions that are obtained using the half datasets. In IMIRS, the resolution evaluation was performed using three steps as described below. From the FSC curve, we used the 0.5 criterion to calculate the reported resolution for the final 3D reconstruction.

(A) Splited the dataset into two parts (splitdataset *.dat out1.dat out2.dat).
(B) Reconstructed the *out1.dat* and *out2.dat* (reconstruct *1.dat).
(C) Calculated the correlation of the two maps with the program *frc*.

### X-ray crystallography

#### Protein expression

The sf21 insect cell line was used to express HEV capsid proteins for crystallization using the detail procedures described below.

(A) The recombinant bacmid DNA obtained using the Bac-to-Bac^®^ Baculovirus Expression System (Version D, Invitrogen) was used to transfect sf21 cells.
(B) The P1 viral stock was harvested by centrifuging down the cell debris at 3000 *g* for 15 min. The cell pellet was then lysed to detect the presence of recombinant proteins by SDS-PAGE gels. The P1 viral stock was then amplified twice to generate a P3 viral stock with higher titer.
(C) For recombinant protein expression, sf21 cells were mixed with 10% of the P3 viral stock and incubated for 48 h at 27 °C postinfection. The harvested insect cell pellet was sonicated in lysis buffer. The lysate was clarified by centrifugation at 25,000 *g* for 30 min.
(D) His-tagged capsid protein of HEV (HEV-CP) was purified using a Ni–NTA column, an anion-exchange (Q) column and a gel-filtration column. The gel-filtration chromatogram indicated that HEV-CP was eluted as homo-dimers.
(E) The final purified HEV-CP was at least 95% pure as indicated by SDS-PAGE. Approximately 25 mg of purified HEV-CP could be obtained from each liter of sf21 cells.

#### Crystallization

**Fig. 9:**
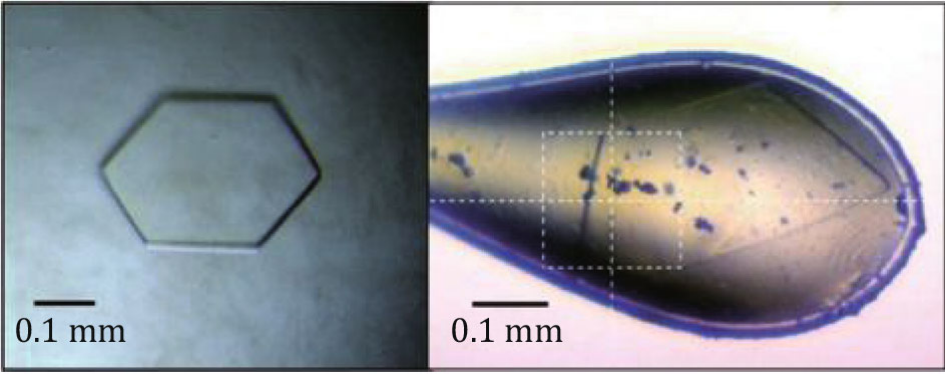
Optimized HEV crystals. *Left*: an optimized crystal as the result of crush seeding and detergent addition. *Right*: a crystal in the cryo-loop taken during data collection

(A) To search for crystallization conditions, an automated robot (Hydra II plus) and 96-well screen trays were used for high throughput screening. Many solvent conditions were tested using commercial screening kits including those from Qiagen (AmSO4, Classics, Classics-Lite, pHClear I & II, MbClass I & II, MPD, Anions, Cations, PEG, and Cryos) and Hampton Research (Index HT, Crystal Screen HT, and SaltRX).
(B) The initial crystallization condition was optimized by varying several factors including pH, temperature, precipitant or protein concentration, precipitant type, volume ratio between protein and mother liquor, and the size of the hanging drop. Microseeding and the addition of the detergent *n*-tetradecyl-b-D-maltoside accelerated crystal growth and improved crystal quality.
(C) For data collection, HEV-CP crystals were flashed frozen in liquid nitrogen in cryo-protectant made of the mother liquor supplemented with 20% of glycerol (Fig. 9).

#### Data collection for HEV crystals

The large unit cell dimensions of the HEV-CP crystals suggest the formation of VLPs during crystallization. Our data collection was performed at the Advanced Photon Source (APS) (Argonne National Laboratory, Lemont, IL) on beamline SBC-19-ID with a wavelength of 0.97934 Å using a 3 × 3 mosaic CCD detector (ADSC Quantum 315).

(A) Exposure time. With intense and focused synchrotron radiation, an exposure time of only a few seconds is usually sufficient. HEV-CP diffraction data were collected using a 10 s exposure time.
(B) Detector distance at APS. A 400-mm detector distance was sufficient to produce well-resolved diffraction spots to 3.5-Å resolution.
(C) Oscillation angle. Preliminary test shots showed our HEV crystals had a high mosaicity (0.6–1.2). While the use of large oscillation angels can help to collect whole reflections, reflection overlapping becomes a serious problem. In our case, a 0.5° oscillation angle produced balanced results.
(D) Oscillation range. In order to determine how many degrees of data collection were needed for a complete dataset, a few test images (usually with oscillation angles of 0°, 45°, and 90°) were taken to check the space group of the crystal.

**Table 1:** Diffraction data statistics

Data collection	
Space group	P6_3_
Unit cell dimension (Å)	*a, b* = 241.1; *c* = 519.9
Resolution (Å)	60–3.5
Total number of frames	239 from two crystals
Total number of reflection	5,236,044
Unique reflection	214,958
*I*/*σ*	11.5 (2.9)
Redundancy	6.9 (6.5)
Completeness (%)	93.7 (92.8)
*R*_merge_	20.9 (67.6)

With many screened crystals (60–70) in total and all randomly oriented), we were able to collect several full data sets with good mosaicity and completeness. The X-ray diffraction data was summarized in Table 1.

#### Diffraction data processing

The observed diffraction data were recorded as raw image files (*.img* or *.osc* files). These data must be indexed, integrated, and scaled in order to obtain their reciprocal space indices (h, k, l) and their corresponding diffraction intensity (*I*) over the estimated error in intensity (*σ*). HEV-CP diffraction data were processed using HKL2000 (Otwinowski and Minor 1997), which consists of XdisplayF, Denzo, and Scalepack.

**Fig. 10:**
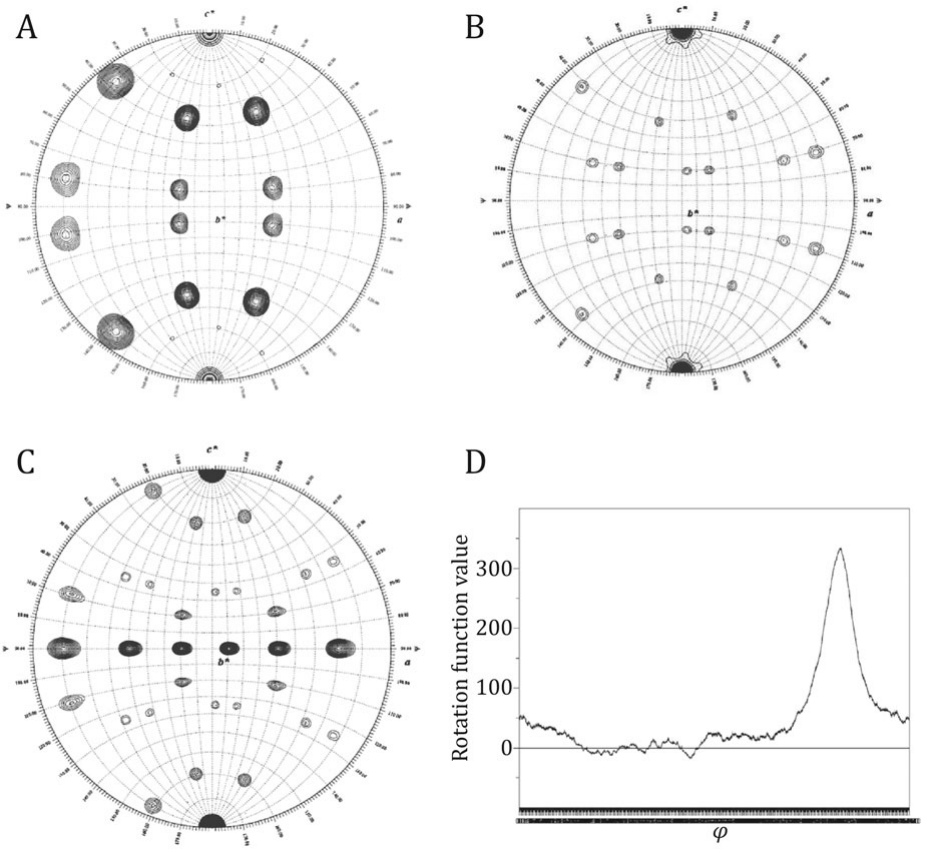
The GLRF self-rotation search results for the crystallographic data. A–C Fast GLRF shows the symmetry solutions around five-, three-, and two-fold axes. **A** *κ* = 72°, **B** *κ* = 120°, **C** *κ* = 180°. **D** Slow GLRF is used to determine the exact value for *φ* angle, by fixing *κ* = 72° and *ψ* = 142.62°. The result indicates *φ* = 49.00°
(B) The exact positions of the symmetry axes were fine-tuned by carrying out a series of slow self-rotation searches using a smaller search interval (e.g., 0.2°) (Fig. 10D). Since the three-fold NCS axis of the HEV-VLP is parallel to the crystallographic 6_3_ axis, there is only one degree of freedom—rotation about the crystal 6_3_ axis.
(C) The difference between our computationally determined *φ* angle and the *φ*0 angle of an icosahedron at the standard 222 orientation, ∆*φ*, defines the actual orientation of our HEV-VLP relative to that of a standard icosahedron. Because all symmetry axes are related by fixed angles in an icosahedron, *φ* can be accurately computed from the large numbers of symmetry axes presented in an icosahedron. The averaged angle for ∆*φ* was determined to be 49.00°. Knowing how the crystallographic VLP sits in the unit cell allowed us to relate this orientation to the EM standard orientation (Fig. 11 *Left*)—this angle, when expressed in Eulerian angles, was *α* = 49.000°, *β* = –20.905°, *γ* = 0.000° (Fig. 11 *Right*).

To orient the HEV-VLP into the crystal unit cell, we performed a self-rotation search using the program GLRF (Tong and Rossmann 1997) as described below. The solution of the GLRF is expressed in polar angles (*φ*, *ψ*, *κ*), and rotation symmetries can be found when *κ* is equal to 180^o^, 120^o^, and 72^o^, corresponding to the icosahedral two-fold, three-fold, and five-fold symmetry.

(A) Two-dimensional searches (*φ* and *ψ* = 0–180°) were performed by fixing *κ* at 180°, 120°, and 72°. The solutions identifying the positions of all the NCS elements expected for an icosahedral particle (Fig. 10A–C).

**Fig. 11:**
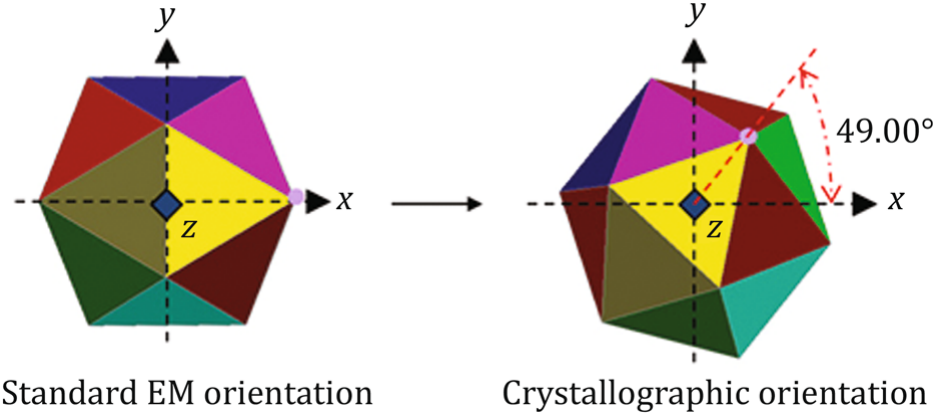
Rotation of the HEV density map. *Left*: the cryo-EM density map orientation. *Right*: the crystallographic orientation as determined by GLRF
(D) Considering the unit cell dimensions and the size of the VLP, there should be one VLP in each unit cell. The particle should sit on the *c*-axis, but its *z* coordinate is arbitrary in the space group P63. For convenience, we fixed the particle center at the origin (*x* = 0, *y* = 0, *z* = 0).

#### Handling the phasing problem

Our initial phasing model is a 14-Å cryo-EM HEV-VLP reconstructed from the Step 10–20. The EM particle was first rotated from the standard EM orientation by *α* = 49.000°, *β* = –20.905°, *γ* = 0.000° using either MAVE (skew command) or MAPROT (Stein et al. 1994). A mask was then created for the rotated map. The density from the EM map inside the mask (in a P1 cell) was projected into the P63 unit cell using MAVE (expand command). The resulting map, also called the “P-cell map,” showed tight packing of the EM particles. Due to the high symmetry of icosahedral viruses, NCS averaging is a very powerful option for phase improvement and phase extension (Kleywegt and Jone 1996). Our crystallographic asymmetric unit contains 1/3 of the VLP particle, which consists of 20 capsid protein subunits. Therefore, there are a total of 20 NCS operators. Several factors are crucial to a successful phase extension.

(A) Initial resolution. The goal was to maximize the overlap between the crystallographic data set and the EM model (Navaza 2008).
(B) Magnification. It is known that electron microscopy cannot determine the precise scale of the particle—there can be up to ~5% error in the estimation (Dodson 2001).
(C) Orientation. Although the average *φ* angle was determined by GLRF, a small incremental range of *φ* angle can be tested for precise orientation.
(D) Contour level. The importance of defining an appropriate mask around the EM density map cannot be overstated. A high contour level may exclude potential real density, resulting in missing structural information. A low contour level, however, may include excessive noise, introducing errors into phase calculations.

These four parameters can be adjusted to generate the initial phasing model. The quality of the phasing model is assessed primarily by the *R*-factor and correlation coefficient. Phase extension was executed using RAVE (Jones 1992; Kleywegt and Jones 1996) combined with several CCP4 programs (Collaborative 1994). SFALL generated Fcalc from the density map. RSTATS then scaled Fobs and Fcalc together, producing statistics to evaluate the agreement between the two sets of structure factors. With the calculated structure factors, FFT created a 2Fo-Fc map. This new calculated map was averaged by AVE using the 20 NCS operators for 10 cycles. The improved map was then back-transformed to a slightly higher resolution, typically by one-reciprocal lattice point, than at which it was originally calculated. The phase estimates at the extended resolution, though noisy, were fed back into the cyclical procedure, and subsequently refined by NCS averaging. As a result, the phase was gradually extended from low resolution to the desired high-resolution shell. In our studies, phase extension from 14 to 3.5 Å required a total of 123 steps. Our final phase extension yielded an averaging *R*-factor of 31.7 and a correlation coefficient of 79.7 at 3.5-Å resolution.

#### Model building

Our 3.5-Å density map showed highly continuous density with few breaks, prominent side chains for the aromatic residues, and a high percentage of β sheets, consistent with the secondary structure prediction and the characteristics of small RNA viruses. Model building of a single HEV-CP protomer was performed using program O (Jones et al. 1991). In addition, our final 3.5-Å map was sharpened using *B* = −150 Å^2^ to enhance the side chain density. Refinement of the model was performed in CNS (Brunger et al. 1998). Once a monomeric CP model was built, the entire HEV-VLP can then be generated from a single protomer using XPAND (Kleywegt) and MOLEMAN (Kleywegt). Our deposited structure (3HAG) has a Rwork and Rfree of 27.67% and 28.60%.

## Result and discussion

This protocol contains three commonly used methodolgies for solving virus atomic structures: protein expression using baculovirus, single-particle cryo-EM, and X-ray crystallography. Each of them includes multiple intricate steps. Expressing HEV capsid proteins by baculovirus and insect cells helped to bypass the issue of safety and sample shortage. Due to the inherent HEV structural flexibility (as contributed by its flexible surface spikes) and the instrumentation limitation of electron microscope, cryo-EM alone was not able to solve the HEV VLP structure to high resolution. On the other hand, the enormous size of virus particle (typically measured in mega Dalton) makes particle crystallization, X-ray diffraction data collection and processing very challenging.  Thus, solving virus structure with X-ray crystallography alone has been shown to be a difficult endeavor.  Fortunately, using the low resolution cryo-EM reconstruction as the initial phase model and combining it with X-ray diffraction data by taking advantage of averaging electron density maps of the highly symmetrical virus particle, we were able to determine the HEV VLP structure to 3.5 Å resolution. These combined approaches can be implemented to solve the atomic structures of other medically significant viruses.

Our VLP structure is the first high-resolution structure of HEV viral particle and reveals a number of details different from those of calicivirus to which HEV was previously classified. First, the three domains S, P1, and P2 are arranged in a linear sequence in HEV-CP. Second, P1 domain adopts a different protein fold, which forms trimeric protrusions. These structural details provide an important framework for better understanding of the assembly of hepatitis E virus and insights into finding potential antiviral targets to combat this virus.
